# Harnessing a Feasible and Versatile *ex vivo* Calvarial Suture 2-D Culture System to Study Suture Biology

**DOI:** 10.3389/fphys.2022.823661

**Published:** 2022-02-10

**Authors:** Natalina Quarto, Siddharth Menon, Michelle Griffin, Julika Huber, Michael T. Longaker

**Affiliations:** ^1^Hagey Laboratory for Pediatric Regenerative Medicine, Stanford University School of Medicine, Stanford, CA, United States; ^2^Division of Plastic and Reconstructive Surgery, Stanford University School of Medicine, Stanford, CA, United States; ^3^Department of Surgery, Stanford University School of Medicine, Stanford, CA, United States; ^4^Dipartimento di Scienze Biomediche Avanzate, Università degli Studi di Napoli Federico II, Naples, Italy; ^5^Institute for Stem Cell Biology and Regenerative Medicine, Stanford University School of Medicine, Stanford, CA, United States; ^6^Department of Plastic Surgery, BG University Hospital Bergmannsheil Bochum, Bochum, Germany

**Keywords:** calvarial suture, *ex vivo* explants, culture method, biology, phenocopy craniosynostosis

## Abstract

As a basic science, craniofacial research embraces multiple facets spanning from molecular regulation of craniofacial development, cell biology/signaling and ultimately translational craniofacial biology. Calvarial sutures coordinate development of the skull, and the premature fusion of one or more, leads to craniosynostosis. Animal models provide significant contributions toward craniofacial biology and clinical/surgical treatments of patients with craniofacial disorders. Studies employing mouse models are costly and time consuming for housing/breeding. Herein, we present the establishment of a calvarial suture explant 2-D culture method that has been proven to be a reliable system showing fidelity with the *in vivo* harvesting procedure to isolate high yields of skeletal stem/progenitor cells from small number of mice. Moreover, this method allows the opportunity to phenocopying models of craniosynostosis and *in vitro* tamoxifen-induction of *Actin^creERT2^;R26^Rainbow^* suture explants to trace clonal expansion. This versatile method tackles needs of large number of mice to perform calvarial suture research.

## Introduction

Calvarial sutures are soft fibrous tissue joining the five flat intramembranous bones of the skull vault (Opperman, 2000; Lenton et al., 2005; Rice, 2008). These fibrous joints consisting of non-ossified mesenchymal cells (suture mesenchyme) are located between the two approaching osteogenic fronts of the skull vault bone. They serve as growth centers playing critical roles in facilitating the expansion and development of the postnatal skull vault in order to accommodate the growing brain (Morriss-Kay and Wilkie, 2005; Nieman et al., 2012). Early fusion of the cranial sutures leads to a pathological condition known as craniosynostosis (Wilkie, 1997; Wilkie and Morriss-Kay, 2001; Senarath-Yapa et al., 2012; Wilkie et al., 2017).

The suture mesenchyme has been defined as a niche of stem cells essential for calvarial morphogenesis and injury repair (Zhao et al., 2015; Doro et al., 2017). Our recent work has identified a skeletal stem/progenitor cell population resident within the cranial sutures, and has preeminently unveiled the importance of a proper balance of this skeletal stem/progenitor cell population representation within the suture mesenchyme for the maintenance of suture patency (Menon et al., 2021). Moreover, a decrease in representation of this skeletal stem/progenitor cell population may account for cranial suture closure and craniosynostosis (Menon et al., 2021).

Studies focused on *in vivo* isolation and in-depth analysis of cells from calvarial sutures, like our previous study, require large number of mice in order to gain substantial cell-yields sufficient to perform experiments on isolated cells (Menon et al., 2021). This demand is indeed costly, time consuming and a major limitation to these studies. Therefore, to overcome this hurdle we thought to develop an alternative procedure harnessing a significantly reduced number of mice, and yet valuable when compared to *in vivo* experimental procedures.

The purpose of this article is to present our *ex vivo* calvarial suture explant 2-D model as a feasible alternative to *in vivo* methods. Herein, we describe three experimental advantages provided by this methodology: (1) High yields of skeletal stem/progenitor cells isolated; (2) Phenocopying models of craniosynostosis; (3) *In vitro* tamoxifen induction of *Actin^creERT2^;R26^Rainbow^* suture explants to trace clonal expansion.

We believe our method will be of interest and useful to craniofacial biology researchers investigating calvarial suture.

## Results

### Experimental Design

In the first part of this manuscript, we will describe the preparation of calvarial sutures explanted from postnatal (pN) day 3 and 15 mice, their 2-D *in vitro* growth and the isolation of a skeletal stem/progenitor cells previously isolated from *in vivo* calvarial sutures (Menon et al., 2021).

### Suture Explants Preparation

**FIGURE 1:**
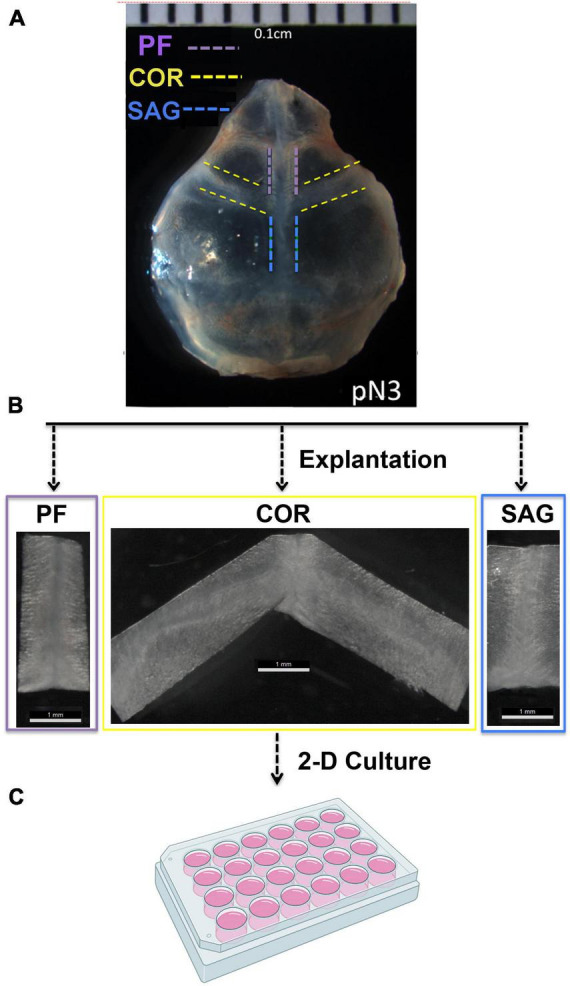
Outline of the PF, COR, and SAG calvarial suture harvesting. **(A)** Representative phase image of a freshly harvested skull from postnatal day 3 (pN3) CD-1 mouse. Dashed lines mark the prospective area of each calvarial suture to be explanted. **(B)** Explanted PF, COR and SAG sutures. Width ranged from 0.1 to 0.3 cm depending on mice age. Explants from pN3 mice are 0.1 cm approximately. **(C)** 24-multiwell plate used for culturing the explanted sutures as described under the section “Materials and Methods.” Experiments were repeated at least five times.

Posterior frontal (PF), Coronal (COR), and Sagittal (SAG) sutures were explanted from pN3 CD-1 mice (*n* = 20) under a stereomicroscope using a Bard-Parker scalpel (No. 20) (Figure 1). The explanted sutures comprised the suture mesenchyme, osteogenic fronts, overlying pericranium and underlying dura mater tissues. Each harvested suture was individually placed into a 12-multiwell plate and mildly digested with trypsin diluted 1:1 with PBS for 10 min at 37°C. Of note, harvested COR suture explants were divided in two pieces (right and left) and freed of the intersected SAG suture tissue prior digestion, in order to prevent any contamination from the SAG suture. Digestion was neutralized by adding 2 ml of DMEM GlutaMAX medium supplemented with 10% Fetal Bovine Serum, 1% Penicillin/Streptomycin. This mild digestion allowed the explants to spread and the migration of few cells from the edge. After 8 days in culture, and one media change, suture explants were digested with Stem-Pro Accutase (Gibco) at 37°C for 15 min. A gentle digestion with Accutase is a crucial step in order to preserve cell surface antigens probed for in subsequent Fluorescence-Activated Cell Sorting (FACS) procedures. After digestion cells were collected by centrifugation at 1,350 rpm for 15 min and processed for FACS analysis. FACS experiments were performed as described previously (Menon et al., 2021).

**FIGURE 2:**
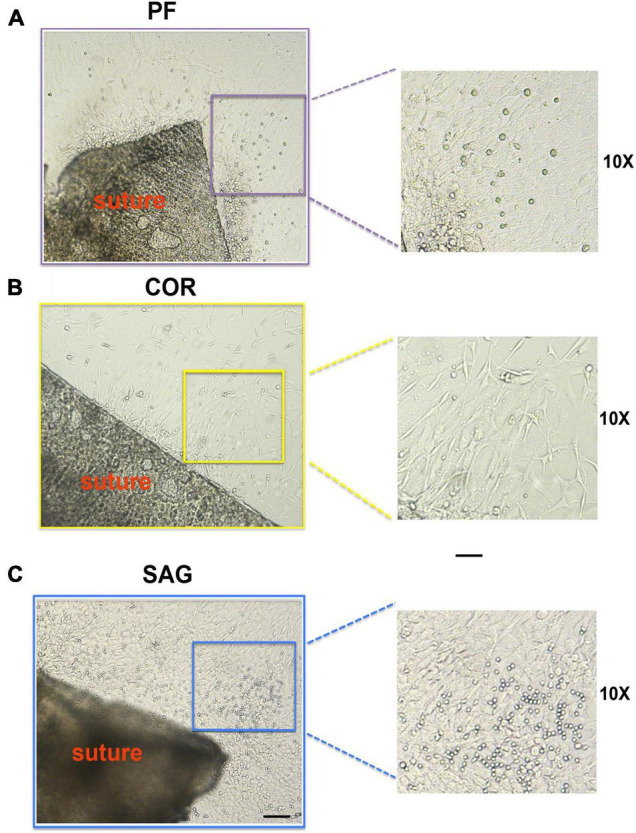
Morphology of cells migrating from PF, COR and SAG suture explants. **(A)** Morphology of cells migrated from the edges of the PF suture explants in culture at day 8. Mix cell morphology with cells showing cuboidal-flat shape, and few other cells with a round or elongated fibroblast-like morphology is observed. **(B)** Cells migrated from COR suture explants display more uniform and elongated fibroblast-like morphology. **(C)** In SAG suture explants culture the migrated cells also show mix morphology, characterized by a larger number of round-shape cells in comparison to PF suture explants. Scale bars value 100 μm.

During the 2-D culturing time, some cells migrated from the sutures explant edges and expanded (Figure 2). Interestingly, these migrated cells showed different morphologies depending on their suture derivation. Cells migrating from PF suture explants displayed mix morphology characterized mainly by a cuboidal-flat shape, and few cells with a round or elongated fibroblast-like morphology (Figure 2A). In contrast, cells from COR suture explants had a more uniform and elongated fibroblast-like morphology (Figure 2B). SAG suture explants culture also showed mix morphology with a larger number of round cells in comparison to PF suture explants (Figure 2C). This differential heterogeneity in cell-morphology may reflect the different embryonic origins of the tissues forming the architecture of each calvarial suture (Jiang et al., 2002; Yoshida et al., 2008). However, we could not rule out that the distinct morphology reflects differences in the extent of cell migration and/or proliferation.

### Side-by-Side Comparison of Skeletal Stem/Progenitor Cells Isolated From *ex vivo* Calvarial Suture Explants and *in vivo* Calvarial Sutures

**FIGURE 3:**
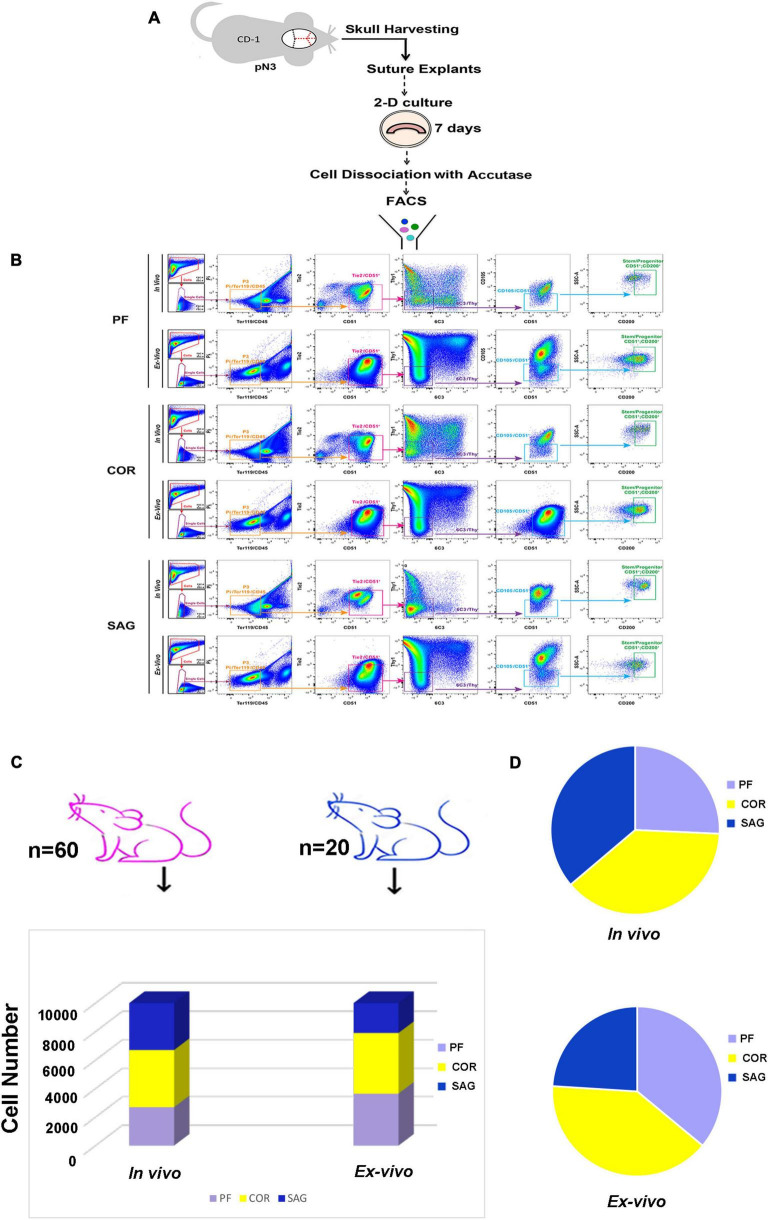
Side-by-side comparison of FACS profiles of skeletal stem/progenitor cells isolated from *in vivo* calvarial sutures and *ex vivo* 2-D suture explants. **(A)** Schematic outline of the experimental procedure. **(B)** Representative FACS plots of skeletal stem/progenitor (CD51^+^/CD200^+^) cells isolated both *in vivo* and *ex vivo* from postnatal day 3 PF (Top panel), COR (Middle panel), and SAG (Bottom panel) sutures. Dissociated cells either from *in vivo* freshly harvest sutures or *ex vivo* suture explants after 8 days in culture were fractionated by side scatter (SSCs) and forward scatter (FSC) to discriminate single cells. Single cells were then fractionated into the P3 population (Pi^–^/Ter119^–^/CD45^–^), and further fractionated by Tie2 and CD51. Tie2^–^/CD51^+^ cell population was gated for Thy1 and 6C3, and the double negative, Thy1^–^/6C3^–^ population was next gated against CD105. Finally, the CD105^–^/CD51^+^ population was gated against CD200 for final isolation of skeletal stem/progenitor cells (CD51^+^/CD200^+^): CD45^–^, Ter119^–^, Tie2^–^, Thy1.1^–^, Thy1.2^–^, 6C3^–^, CD105^–^. **(C)** Histogram showing the high stem/progenitor (CD51^+^/CD200^+^) cell yield from *ex vivo* suture explants system obtained using 20 mice (blue color) versus 60 mice (pink color) needed for the *in vivo* isolation of an equivalent number of cells. **(D)** Cell distribution charts showing the representation of stem/progenitor cells (CD51^+^/CD200^+^) in calvarial sutures *in vivo* and *ex vivo*. Of note, a larger representation of cells are isolated from *ex vivo* 2-D PF and COR suture explants in comparison to *in vivo*, whereas a smaller representation is found in SAG suture explants. Independent *in vivo* and *ex vivo* isolation experiments were performed at least five times.

Having established the *ex vivo* calvarial suture 2-D culture system, we investigated whether this method could be employed to isolate higher or similar yields of skeletal stem/progenitor cells while scaling down the number of mice needed for the *in vivo* isolation procedure. This was accomplished by using the same FACS strategy previously exploited for the *in vivo* isolation of skeletal stem/progenitor cells from the calvarial sutures (Menon et al., 2021). Figure 3A depicts the experimental scheme. We first compared the immune-phenotype of the skeletal cell population isolated from *ex vivo* 2-D cranial suture explants after 8 days in culture, to that of freshly isolated *in vivo*. For this analysis, were utilized twenty mice for the *ex vivo* cell isolation, and sixty for the *in vivo* isolation. Figure 3B illustrates side-by-side FACS profiles of cells isolated from *ex vivo* and *in vivo* sutures. Both, skeletal stem/progenitors isolated either *ex vivo* or *in vivo* shared similar FACS profiles. Of relevance, we obtained an extremely high yield of cells from *ex vivo* suture 2-D culture system using a remarkable smaller number of mice (*n* = 20) in comparison to the *in vivo* (*n* = 60) to isolate an equivalent number of skeletal stem/progenitors (Figure 3C). Moreover, an increase in representation of skeletal stem/progenitor cells in both, *ex vivo* PF and COR suture 2-D explants culture was noticed as compared to the *in vivo* sutures (Figure 3B, left panel, and Figure 3D). Conversely, a decreased representation of skeletal stem/progenitor cells was observed in *ex vivo* SAG suture 2-D (Figure 3B, left panel and Figure 3D).

The representation of skeletal stem/progenitor resident in calvarial sutures declines significantly overtime, from postnatal day 3 (pN) through day pN15 their percentage decrease by 90% in PF suture and approximately 60% in COR and SAG sutures (Menon et al., 2021). Therefore, this severely impacts the yields of skeletal stem/progenitors obtained from *in vivo* isolation using mice older than pN3. A further advantage offered by our *ex vivo* suture 2-D explants culture is the high yield of cells that can be isolated from older mice. As illustrated in Supplementary Figure 1, at pN15 the representation of skeletal stem/progenitors was markedly larger in *ex vivo* 2-D explants culture compared to *in vivo* (Supplementary Figure 1A). This in turn led to higher cell-yields *ex vivo* isolation using only six mice whereas thirty animals were needed to isolate nearly the same number of cells *in vivo* at the pN15 day stage (Supplementary Figure 1B).

The aforementioned advantage provides also the opportunity to isolate skeletal stem/progenitor cells from transgenic mice models of syndromic craniosynostosis such as the *Twist1* heterozygous mouse modeling Saethre-Chotzen syndrome (el Ghouzzi et al., 1997; Carver et al., 2002). Side-by-side isolation of skeletal stem/progenitor cells employing both *in vivo* and *ex vivo* procedure further validated the benefits of our *ex vivo* suture 2-D explants system over *in vivo* isolation procedure (Supplementary Figure 1C).

**FIGURE 4:**
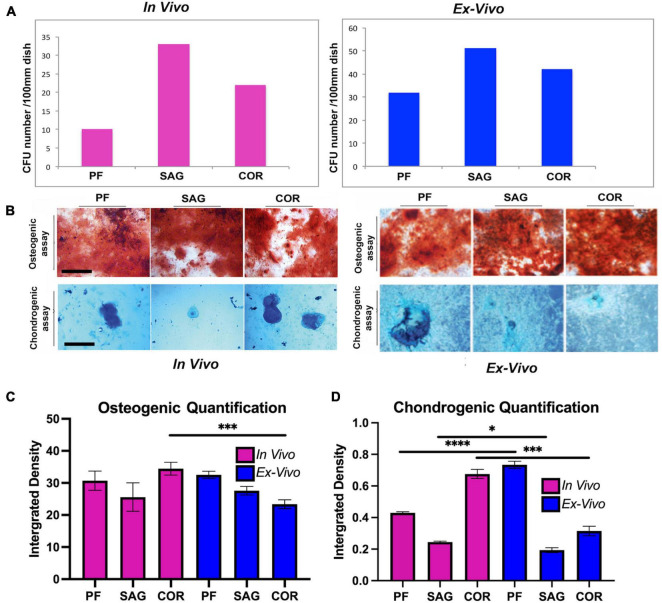
Comparative analysis of CFU formation and lineage specification. **(A)** Colony forming assay (CFU) of skeletal stem/progenitor cells. Graph showing the number of CFUs formed by skeletal stem/progenitor cells isolated *in vivo* from freshly harvested PF, SAG, and COR sutures in comparison to cells isolated from *ex vivo* 2-D PF, SAG and COR suture explants. **(B)** Lineage specification of the two isolated skeletal stem/progenitor cells. (Top panel), Alizarin red staining of cells differentiated toward osteogenic lineage reveals the presence of extracellular matrix mineralization which appears more robust in cells isolated from 2-D suture explants. (Bottom panel), Chondrogenic assay performed on the two skeletal stem/progenitor cells followed by Alcian blue staining to assess proteoglycan production, indicates the chondrogenic specification ability of both cells [*in vivo* data by courtesy, Menon et al. (2021) *Nat Commun.* 12, 4640, doi: 10.1038/s41467-021-24801-6, 2021]. Scale bars value 200 μm. **(C)** Quantification of osteogenic differentiation assay of SSCs isolated from the 3 sutures both *in vivo* and *ex vivo*. Quantification was performed using ImageJ measure tool. Values are an average of at least 3 different fields measured and unpaired students *T-test* statistical analysis was performed. **(D)** Quantification of chondrogenic differentiation assay of SSCs isolated from the 3 sutures both *in vivo* and *ex vivo*. *Ex vivo* 2-D PF suture derived cells differentiate toward the chondrogenic lineage at higher extent than *in vivo* isolated cells. Conversely, *ex vivo* 2-D COR suture derived cells differentiated less than those isolated *in vivo*. Quantification was performed using ImageJ measure tool (ImageJ software program, NIH, Bethesda, MA, United States). Values are an average of at least three different fields measured and unpaired student *T-test* statistical analysis was performed. *P < 0.01, ^***^P < 0.001, ^****^P < 0.0001.

To further compare the skeletal stem cell/progenitor populations isolated with the two different methods, we analyzed their colony forming unit (CFU) capacity and lineage specification. No relevant differences were observed between the two populations, therefore suggesting stringent similarity (Figure 4A). Furthermore, lineage specification assays to determine the ability to differentiate toward osteogenic and chondrogenic lineages highlighted also similar capacities of lineage-specification between *ex vivo* and *in vivo* isolated skeletal stem cells and progenitors (Figure 4B). Of note, the skeletal stem cell/progenitors derived from *ex vivo* PF sutures displayed a higher chondrogenic capacity. This may reflect better *in vitro* adaptation (upon explants culturing) of the cells originally resident in a calvarial suture, such as the PF which undergoes to fusion through an endochondral differentiation program (Sahar et al., 2005). In contrast, cells derived from *ex vivo* COR sutures differentiated less toward the chondrogenic lineage as compared to those isolated from *in vivo*. Quantification of alizarin red and alcian blue staining are illustrated in Figures 4C,D, respectively.

### *Ex vivo* and *in vivo* Skeletal Stem/Progenitor Cells Transcriptomic Profiles Cross-Referencing

**FIGURE 5:**
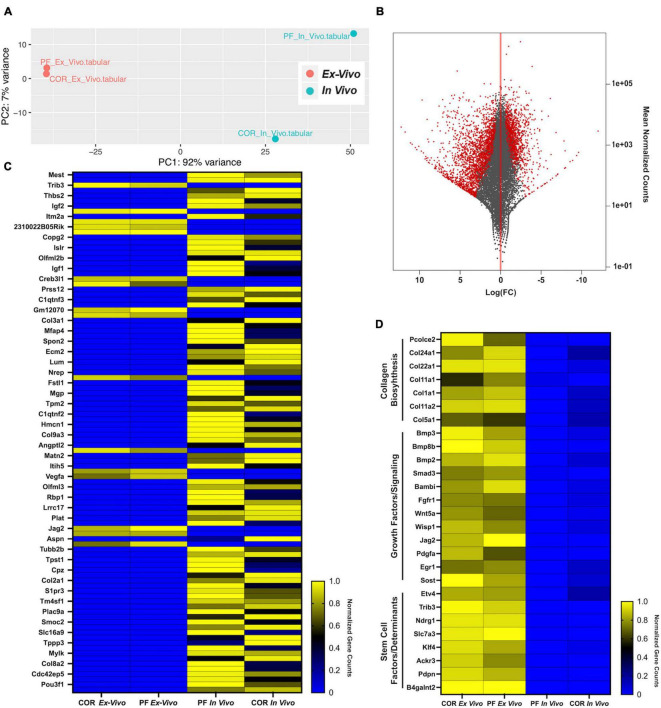
Transcriptomic cross-referencing of skeletal stem/progenitor cells from *ex vivo* 2-D suture explants to *in vivo* cells. **(A)** Principal component analysis of skeletal stem/progenitor cells isolated from *in vivo* and *ex vivo* PF and COR sutures from pN3 mice. PC1 showed a 92% variance and PC2 a 7% variance among the two groups *in vivo* and *ex vivo*. **(B)** MA plot of Log (FC) vs. mean of normalized counts showing distribution of all genes, *in vivo* vs. *ex vivo* highlights a greater distribution of upregulated genes *in vivo* (red, significant). **(C)** Heatmap representing normalized gene counts for the top 100 ranked genes from differential expression analysis between skeletal stem/progenitor cells isolated from *in vivo* and *ex vivo* mouse calvarial sutures (Blue, downregulated; Yellow, upregulated). **(D)** Heatmap representing normalized gene counts for genes of interested upregulated in *ex vivo* skeletal stem/progenitor cells (Blue, downregulated; Yellow, upregulated).

To further assess the similarities and differences between skeletal stem/progenitor cells isolated from the calvarial sutures in both *ex vivo* and *in vivo* conditions, we performed bulk RNA-sequencing transcriptomic analysis of skeletal stem/progenitor cell isolated from PF and COR sutures. We reasoned to choose for this analysis a fusing homotypic suture (formed exclusively by neural crest derived tissue origin) such as, the PF suture, and the COR patent heterotypic suture formed by mix neural crest and paraxial-mesoderm derived tissue origin, with suture mesenchyme of paraxial-mesoderm tissue derivation (Jiang et al., 2002). Analysis was performed using the DESeq2 tool on the Galaxy platform (Afgan et al., 2018). Principal Component (PC) analysis demonstrated 91% variance in PC1 and 7% in PC2 between the *ex vivo* and *in vivo* conditions (Figure 5A). In contrast to *in vivo* isolated skeletal stem/progenitor cells, PC analysis showed tight clustering between skeletal stem/projector cells isolated from *ex vivo* sutures, while those isolated *in vivo*, were more dispart (Figure 5A) of note, this PCA clustering implies that the skeletal stem/progenitor cells isolated from the two sutures become more transcriptionally similar *ex vivo* than *in vivo.* This finding would suggest that our system could potentially underscore transcriptomic difference existing *in vivo* between skeletal stem/projector cells isolated from different calvarial sutures. MA plot of log (FC) vs. mean of normalized counts showed a greater distribution of statistically significant genes upregulated in the *in vivo* vs. *ex vivo* condition, red color indicates significance (Figure 5B). Next, we analyzed the top ranked upregulated and downregulated genes from our differentiation expression analysis. Figure 5C is a heatmap of normalized gene count for the top 100 ranked differentially expressed genes from this analysis. Interestingly, among the genes found upregulated in *ex vivo* skeletal stem/progenitor cells were genes playing cell stemness function such as *Trib3*, which confers cell stemness through interaction with beta-catenin and by enhancing *Sox2* transcription (Yu et al., 2019; Zhang et al., 2019; Lu et al., 2020), *Slca3* gene, a component of a “consensus gene signature” defining self-renewal of stem cells (Kim et al., 2014) and Ndrg1r a hypoxia induced gene promoting stem-like properties were also upregulated (Wang et al., 2017). Several others genes playing similar function such as *Pdpn, Egr1*, *Etv4*, and *Ackr/CXCR7* were unregulated in *ex vivo* skeletal stem/progenitor cells as well (Merino et al., 2015; Sakakini et al., 2016; Tang et al., 2016; Park et al., 2017; Danielyan et al., 2020; Zhu et al., 2021). In addition, genes encoding growth factors, signaling components, masters of collagen biosynthesis (Pcolce2) (Baicu et al., 2012) and several collagen proteins as well were also part of the upregulation profile (Figures 5C,D).

Of relevance, *Mest1* was the only gene with functional regulatory stemness property (Hasegawa et al., 2020), identified from the analysis of genes downregulated in *ex vivo* skeletal stem/progenitor cells, and interestingly *Copg2* a member of mouse imprinted genes linked to *Mest1* was also downregulated (Lee et al., 2000; Figure 5C). Along with aforementioned, several genes encoding cell-adhesion proteins were also noticed, among them were *Spon2* (Zhang et al., 2018), *Smoc2* (Morkmued et al., 2020). Furthermore, SLR Immunoglobulin Superfamily Containing Leucine Rich Repeat (*Islr*), a secreted stromal protein promoting intestinal regeneration through inhibition of hippo signaling (Xu et al., 2020) was also downregulated (Figure 5C).

### Phenocopying Models of Craniosynostosis

**FIGURE 6:**
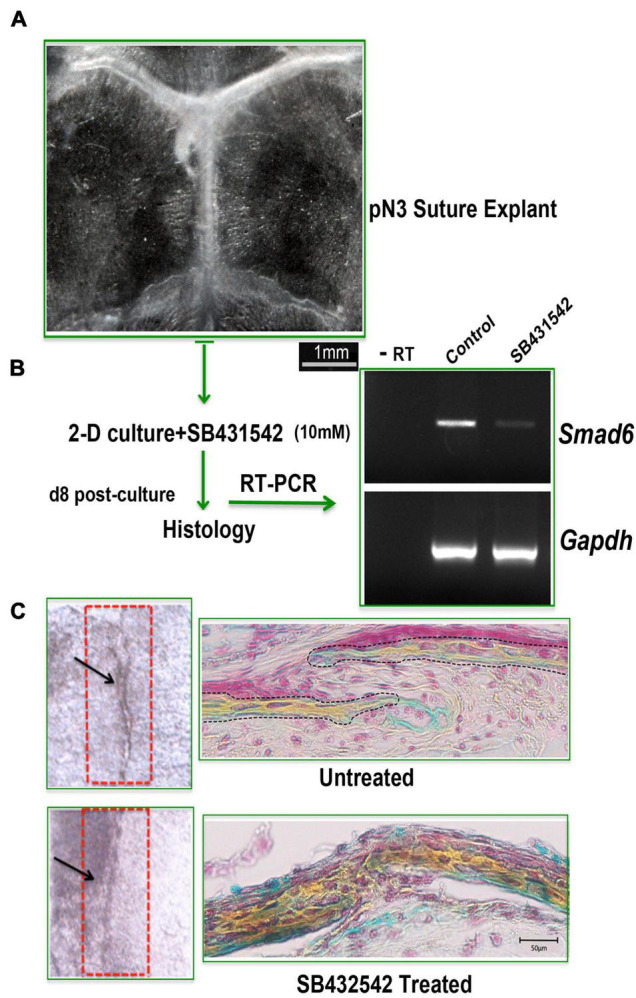
2-D suture explants as tool for phenocopying non-syndromic craniosynostosis. **(A)** Representative image of a freshly explanted SAG suture from pN 3 CD-1 mouse. Explants were cultured for 8 days with or without SB432542 (10 mM). **(B)** RT-PCR analysis of *Smad6* expression level to assess the effectiveness of SB432542 treatment. **(C)** (Left panel) Whole mount top view of untreated and SB431542-treated SAG suture explants with black arrows marking the midline of the SAG suture which appears fused in SB431542-treated explants. (Right panel) Movat’s pentachrome staining of coronal sections of suture explants reveals lack of suture mesenchyme in SB431542-treated SAG suture with bony tissue replacing the suture mesenchyme and fusion [histology data by courtesy, Menon et al. (2021) *Nat Commun* 12, 4640, doi: 10.1038/s41467-021-24801-6, 2021].

Craniosynostosis is a pathologic craniofacial disorder defined as the premature fusion of one or more calvarial sutures. Our recent work has revealed the importance of a proper balance of skeletal stem/progenitors resident in the calvarial suture to maintain patency, and that a dysregulation of this balance may underlay to craniosynostosis. Herein, we show that our *ex vivo* suture explant 2-D culture is a suitable tool to model craniosynostosis (Menon et al., 2021). Figures 6A–C illustrates an experiment performed using *ex vivo* SAG suture explants in 2-D culture to phenocopy a *de novo* and transmitted mutations in inhibitory SMAD6 (I-SMAD6) gene leading to SAG suture craniosynostosis in humans (Timberlake et al., 2016).

The phenocopying of SAG suture craniosynostosis described above was achieved by inhibiting TGFβ signaling with the small molecule SB431542. The rationale behind our experimental approach is supported by the knowledge that inhibition of endogenous TGFβ by using SB431542 triggers down regulation of I-SMAD6, therefore leading to enhanced activation of BMP signaling and induction of osteogenesis (Maeda et al., 2004). After 8 days in culture, SAG suture explants treated with SB431542 showed a sharp downregulation of *Smad6* expression as compared to untreated explants (Figure 6B) and fused (Figure 6C, bottom panel). In contrast, untreated suture explants remained patent and the suture mesenchyme was still present (Figure 6C, top panel).

### *In vitro* Tamoxifen Induction of *Actin^creERT2^;R26^Rainbow^* Suture Explants to Trace Cell Clonal Expansion

**FIGURE 7:**
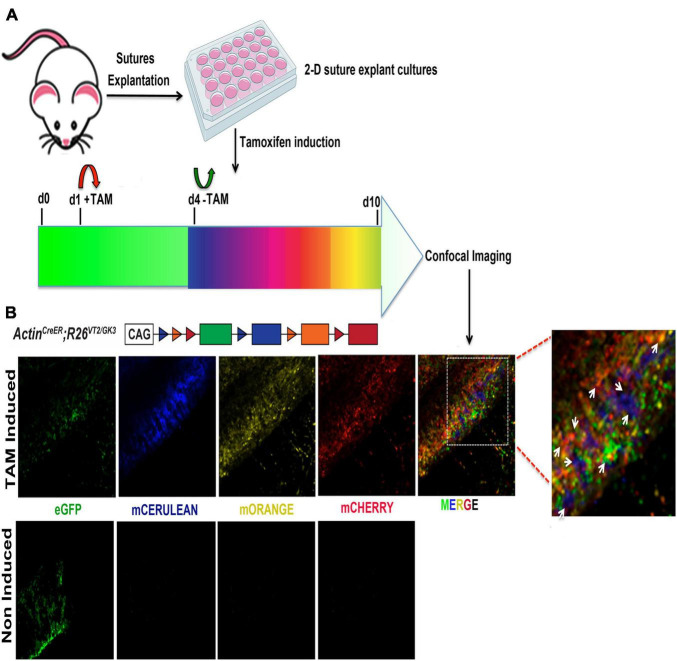
Clonal induction of Rainbow 2-D suture explants. **(A)** Schematic representation of the experimental procedure followed for the tamoxifen (TAM) induction of 2-D suture explants derived from Rainbow mice (Ueno and Weissman, 2006). Upon activation of Cre recombinase by TAM individual cells randomly and permanently express one of four fluorescent protein (FP) colors (cytoplasmic green—eGFP, blue—mCerulean, membrane yellow—mOrange and membrane red—mCherry) **(B)** Confocal imaging of 2-D SAG suture explants. Randomization of color-coding in activated Rainbow 2-D SAG explants shows equal distribution of each fluorescent-labeled cell color (eGFP, mCerulean, mOrange, and mCherry) at day 10 culture. Individual channels are (left side), merged (right side) with white arrows indicating clones.

The use of transgenic mice for lineage tracing and genetic manipulation through targeted tamoxifen activation of an inducible Cre-LOX system is a powerful tool commonly implemented in many *in vivo* animal studies. Herein, we tested the feasibility of *in vitro* tamoxifen induction of *Actin^creERT2^;R26^Rainbow^ ex vivo* suture explants 2-D culture. Rainbow mice have been widely utilized for tracing clonal expansion of individual cells in several systems (Ueno and Weissman, 2006; Chan et al., 2015; Ransom et al., 2018; Foster et al., 2020). Upon activation of Cre recombinase by tamoxifen (TAM), individual cells express randomly and permanently one of four fluorescent protein (FP) colors (cytoplasmic green—eGFP, membrane red—mCherry, membrane yellow—mOrange, and membrane blue—mCerulean). This allows the visualization of clonal expansion of cells of interest as continuous regions of single colors. We have previously employed rainbow mice for *in vivo* clonal analysis of the calvarial sutures. Herein, we tested the feasibility of *in vitro* tamoxifen induction of *Actin^creERT2^;R26^Rainbow^ ex vivo* suture explants 2-D culture to trace cell clonal expansion. Figure 7A illustrates a schematic of the experimental procedure performed for the *in vitro* TAM induction of 2-D suture explants. Confocal imaging of TAM treated 2-D SAG suture explants, at day 10 in culture, identified the presence of cell-clonality as marked by single colors (Figure 7B, top panels). Non-induced explants did not show signals (Figure 7B, bottom panels). These results indicate that the *ex vivo* calvarial suture explants system is suitable and feasible for TAM and/or other chemically induced CRE-LOX recombination systems managing reporter expression and/or gene activation/inactivation, and therefore to perform *in vitro* analysis of clonal expansion. Furthermore, this finding provides the opportunity to screen potential effects that factors and/or drugs may trigger either on the growth or fate of a calvarial suture.

## Discussion

We have established a feasible procedure providing several advantages suitable for researchers in the field of calvarial suture biology and skeletal stem/progenitor cells as well. With its relative simplicity and experimental feasibility this *ex vivo* 2-D calvarial suture explants method will overcome the technical hurdle of *in vivo* isolation such as cell yields, time consuming to harvest sutures, breeding mice, and most importantly, monetary cost. This method allows for isolating high yields of cells circumventing needs of a large number of animals as needed for the *in vivo* isolation. Importantly, cells isolated using this procedure share the same immunophenotype FACS profile, CFU potential and lineage fate specification with cells isolated *in vivo* from freshly harvested sutures, thus recapitulating an *in vivo* isolation outcome. These aforementioned findings indicate that *ex vivo* 2-D calvarial suture explant method preserves the integrity of skeletal stem/progenitor cells “niche.” Moreover, the finding stemming from the comparative analysis of skeletal stem/progenitor cells isolated *in vivo* and *ex- vivo*, revealing a higher number of PF and COR suture-derived cells isolated from *ex vivo* 2-D culture explants in comparison to *in vivo*, also suggest an enrichment of the skeletal stem/progenitor cells “niche” (Figures 3A,D). Thus, it appears that the two suture-derived cell-populations benefit from the timing in 2-D culture, and therefore expand. An explanation for this finding could be found in the tissue composition of PF and COR sutures, comprising osteogenic fronts (two in the PF suture and one in COR suture) of the frontal bones which, elaborate several factors included FGF ligands inducing cell proliferation (Quarto et al., 2009; Li et al., 2010). Therefore, it is likely that during the timing in culture secreted growth factor such as, FGF-2 could trigger proliferation of the skeletal stem/progenitor cells and thereby their enrichment.

Data presented in the first part of this study established a stringent correlation between *ex vivo* and *in vivo* skeletal stem/progenitor cells at level of immunophenotype FACS profile, CFU capacity and lineage specification. Despite these similarities, we sought to further investigate these cells at a different level analyzing their transcriptomic profiles. Therefore, we cross-referenced transcriptomic *ex vivo* and *in vivo* skeletal stem/progenitor profiles. Principal component analysis (PCA) revealed that transcriptomic divergences observed between *in vivo* PF and COR suture-derived skeletal stem/progenitor cells were narrowed in the corresponding *ex vivo* skeletal stem/progenitor cells. The closer transcriptomic landscape profile found between *ex vivo* PF and COR-derived skeletal stem/progenitor may represent an outcome of cell-adaptation to a standardized/uniform culture environment, this finding suggests less heterogeneity between *ex vivo* skeletal stem/progenitor cells derived from PF and COR sutures in comparison to the corresponding two populations isolated *in vivo*. Differential gene expression analysis, highlighted upregulation of several interesting genes in the *ex vivo* skeletal stem/progenitor cells. Among them, the procollagen C-endopeptidase enhancer (PCOLCE), an extra-cellular matrix (ECM) remodeling molecule involved in collagen biosynthesis (Baicu et al., 2012), and a repertoire of genes encoding collagen proteins components of the ECM. Of relevance, several genes encoding growth factors and stem cells factors/determinants controlling stemness (Kim et al., 2014; Kuruvilla et al., 2015; Ghaleb and Yang, 2017; Park et al., 2017; Yu et al., 2019; Zhang et al., 2019; Lu et al., 2020; Zhu et al., 2021), self-renewal (Sakakini et al., 2016) and cell motility/migration (Danielyan et al., 2020; Yu et al., 2021) were also found upregulated significantly in *ex vivo* skeletal stem/progenitor cells. These findings suggest that *ex vivo* skeletal stem/progenitor cells “orchestrate” a compelling repertoire of components known to play an important role for the homeostasis of a stem cell “niche,” the microenvironment maintaining the stem cell in an undifferentiated and self-renewing state (Scadden, 2006; Brizzi et al., 2012; Birbrair, 2017).

Transcriptomic differences observed between *ex vivo* and *in vivo* derived skeletal stem/progenitor cells are not unexpected since they represent an outcome of two different environments. Nevertheless, our *ex vivo* calvarial explant system sustains the biological phenotype of the skeletal stem/progenitor cells. In the light of the transcriptomic data, which have highlighted a marked upregulation of several collagen components of ECM, it is tempting to hypothesize that our *ex vivo* calvarial suture explants likely mimic a 3-D culture condition.

Additionally, data presented herein showed that our system provides the advantage of tackling the need of transgenic mice employed as animal models of human craniosynostosis by phenocopying non-syndromic human craniosynostosis and potentially syndromic craniosynostosis. We have shown that *ex vivo* 2-D culture explants can be successfully employed to phenocopy a non-syndromic human craniosynostosis. Moreover, this system also allows to study genetic syndromic craniosynostosis in mouse models using a significant smaller number of animal and thus, shortening the time needed for breeding and the housing cost.

During the early stages of postnatal development rapid expansion of the skull and brain growth take place, and calvarial sutures finely coordinate these developmental events. During this time, an intrinsic growth (in length) of the calvarial sutures also takes place, as documented by morphometric analysis (Sahar et al., 2005). This growth is coordinated through a complex series of biological events determining the suture fate. Calvarial suture fusion could result from changes in the number of cells in the suture mesenchyme or bone fronts proliferating or undergoing apoptosis. Therefore, rainbow mice may provide the opportunity to visualize through clonal expansion the temporal progression of a calvarial suture (e.g., length) growth as well as its fate. In addition this transgenic mouse represents a system for testing the effect that factors/small molecules may trigger on the growth of cells resident within the cranial suture as well as on patency and/or fusion fates. Our data showed the feasibility of tamoxifen induction of CRE recombination in our *ex vivo* suture explants 2-D culture, allowing for visualization of clonal patterns using a ubiquitous rainbow reporter system.

We believe that the *ex vivo* 2-D calvarial suture explants method presented in this study will enhance our ability to perform studies on calvarial suture biology without demanding a large number of animals and high costs. In conclusion, herein we provide some suitable working templates for analyses of calvarial sutures at different levels spanning from skeletal stem/progenitor cells to craniosynostosis and clonal lineage tracing.

## Materials and Methods

### Animals

Experiments using mice were carried out in accordance with Stanford University Animal Care and Use Committee guidelines. Mice were bred and housed in light, temperature and moisture-controlled Research Animal Facility and were given food and water *ad libitum* in accordance with Stanford University guidelines. CD-1 mice were purchased from Charles Rivers Laboratories Inc., B6 *Twist1*± (Stock# 2221) mice were purchased from The Jackson Laboratory (Bar Harbor, ME, United States). *ActinCreERT2*: Rainbow mice were kindly donated from the laboratory of Dr. Irv Weissman, Institute for Stem Cell Biology and Regenerative Medicine, Stanford University. Genotyping primer sequences and replicons size are listed in Supplementary Table 1.

### 2-D Sutures Explants Preparation and Growth

Mice at day postnatal (pN) 3 and 15 were sacrificed by CO2 asphyxiation and skulls were harvested. Then, calvarial sutures were explanted from skulls under a dissection stereo microscope (Zeiss, Oberkochen, Germany) using a scalpel (Bard-Parker scalpel No. 20 #371620, Aspen Surgical, Caledonia, MI, United States) and the aid of a ruler for accurate measurement. The width of suture explants ranged from 0.1 to 0.3 cm depending on mice age. Each harvested suture was individually placed into a 12-multiwell plate. Then, the suture explants underwent a mild digestion using trypsin diluted 1:1 with PBS for 10 min at 37°C. Digestion was neutralized by adding 2 ml of growth medium made of DMEM GlutaMAX medium supplemented with 10% Fetal Bovine Serum, 1% Penicillin/Streptomycin (Gibco Life Technologies and Invitrogen Corporation, Carlsbad, CA, United States). The 2-D suture explants were maintained in culture for 8 or 10 days depending on experiments.

### Cell Dissociation of 2-D Calvarial Suture Explants

After the appropriate time in culture each suture explant was digested with 160 μl of Stem-Pro Accutase (Gibco) at 37°C for 15 min. Digestion was neutralized by adding 320 μl of DMEM GlutaMAX medium supplemented with 10% Fetal Bovine Serum, 1% Penicillin/Streptomycin. Cells released from each suture explant were pooled, collected by centrifugation at 1,350 rpm for 15 min and processed for FACS analysis.

### Fluorescence-Activated Cell Sorting

Fluorescence-Activated Cell Sorting (FACS) procedure was performed as previously described (Menon et al., 2021) using the FACS Aria II in the Lorey Lokey Stem Cell Institute Shared FACS Facility. Briefly, hematopoietic (CD45^+^) and dead cells (Pi^+^) were gated out and the remaining population (P3) was fractionated based on the following surface antigens as previously described (Menon et al., 2021). Skeletal stem/progenitor cell (CD51^+^;CD200^+^): CD45^–^, Ter119^–^, Tie2^–^, Thy1.1^–^, Thy1.2^–^, 6C3^–^, CD105^–^, CD51^+^, CD200^+^. Highly pure, double sorted skeletal stem/progenitor cells were either sorted directly into TRIzol Reagent (Ambion-Life Technologies, Carlsbad, CA, United States) for RNA isolation or alpha-MEM supplemented with 10% fetal bovine serum, 1% penicillin–streptomycin (Gibco-Life Technologies, Grand Island, NY, United States), and 0.1% ciprofloxacin HCl (bioWORLD, Dublin, OH, United States) for cell culture and differentiation assays. Experiments were performed at least five times. Antibodies and media used for FACS isolation of skeletal stem/progenitor cells are listed in Supplementary Tables 2, 3.

### Colony-Forming Units Assay

Skeletal stem/progenitor cells colony forming unit capacity was assessed as previously described (Menon et al., 2021). Briefly, 500 freshly isolated skeletal stem/progenitor cells from pN3, CD-1 mice PF, SAG, and COR sutures using FACS procedure, were seeded into a 10 cm^2^ plate pre-coated with 0.1% gelatin (EmbryoMax, Millipore, Burlington MA, United States) in alpha-MEM GlutaMAX (supplemented with 10% fetal bovine serum 1% penicillin–streptomycin (Gibco-Life Technologies, Grand Island, NY, United States), and 0.1% ciprofloxacin HCl (bioWORLD, Dublin, OH, United States). Cells were incubated under low O_2_ conditions (2% atmospheric oxygen, 7.5% CO_2_). After 2 weeks, evaluation of CFU-colonies was assessed by crystal violet staining. Cell colonies were imaged under an automated inverted microscope Leica DMI 4000B (Leica, Buffalo Grove, IL, United States). Colonies with >50 cells or more were counted. Additional information regarding reagents used refer to Supplementary Table 3.

### Differentiation Assays

Osteogenic and chondrogenic lineage specification was assessed using the Stem Pro osteogenesis or chondrogenesis differentiation kit (Gibco-Life Technologies, Grand Island, NY, United States). Freshly sorted skeletal stem/progenitor cells were seeded into a 96-well plate, pre-coated with 0.1% gelatin (EmbryoMax, Millipore, Burlington MA, United States) and seeded with a density of 3 × 10^3^ cells/well for osteogenic differentiation assays. For chondrogenic differentiation assays, 1 × 10^3^ cells/well were seeded into a 96-well plate. Upon cell-confluence, growth media was replaced with Stem Pro osteogenic/chondrogenic media (Gibco A10069-01, Grand Island, NY) according to the manufacture’ instructions. Media was changed every other day for 21 days (osteogenic) or 40 days (chondrogenic). Osteogenic differentiation was assessed by alizarin red staining and chondrogenic by alcian blue staining as previously described (Quarto et al., 2010, 2012). For information regarding reagents used refer to Supplementary Table 3. Osteogenic and chondrogenic linage differentiations were quantified using ImageJ (NIH, v2.0) image analysis software. In brief, the measure tool was used to measure integrated density within an ROI, values are an average of at least 3 different fields measured. Unpaired students *T-test* statistical analysis was performed on PF *In vivo* vs. PF *Ex vivo*, SAG *In vivo* vs. SAG *Ex vivo* and COR *In vivo* vs. COR *Ex vivo.*

### 2-D Calvarial Suture Explants SB431542 Treatment

For SB431542, treatment, sutures freshly explanted from pN3 CD-1 mice were incubated in DMEM GlutaMAX medium supplemented with 10% Fetal Bovine Serum, 1% Penicillin/Streptomycin and addition of 10 μM SB431542 (SelleckChem.Com # S1067, Houston, TX), or DMSO vehicle (for the control untreated sutures). Media was changed every 2 days. After 8 days in culture sutures were processed as described above and cells submitted for FACS analysis. For information regarding reagents used refer to Supplementary Table 4.

### RT-PCR Analysis for Genes Expression

To analyze the expression level of *Smad6*, RNAs were isolated from SB431542 treated and untreated suture explants (*n* = 6/each group) by homogenization in 0.6 ml of TRIzol (Ambion-Life Technologies, Carlsbad, CA, United States) using a Pellet Pestle Motor Kontes (# 3411E25, DWK Life Sciences Kimble, Thermo Fisher Scientific) (Sahar et al., 2005). Isolated RNAs were submitted to RT-PCR procedure as previously described (Quarto et al., 2012). Primer-sequences for *Smad6* and *Gapdh* genes, and PCR conditions are reported in Supplementary Table 1. All additional reagents used are listed in Supplementary Table 4.

### Histology

2-D SAG suture explants were harvested and fixed overnight at 4°C, in 4.0% PFA (Electron Microscopy Sciences, Hatfield, PA, United States) and decalcified in 19% EDTA at 4°C for 1 day. Then specimens were soaked in 30% (mass/vol) sucrose in PBS at 4°C for 24 h and embedded in Tissue Tek O.C.T. (Sakura Finetek, Torrance, CA, United States). Specimens were cut in 10 μm sections, and stained with a modified Movat’s Pentachrome procedure (Russell, 1972). Histological sections were examined with a Carl Zeiss Axioplan 2 (Zeiss, Oberkochen, Germany) microscope. Images were acquired by AxioVision (Zeiss, Oberkochen, Germany) and combined by Adobe Photoshop (Adobe Systems). Results were obtained from at least two animals and staining was carried out in triplicate.

### Bulk RNA Sequencing

Bulk RNA sequencing was performed as previously described (Menon et al., 2021). Briefly, skeletal stem/progenitor cells isolated from the PF and COR sutures *in vivo* at pN3 CD-1 mice and *ex vivo* PF and COR explants were double sorted directly into TRIzol Reagent (Ambion-Life Technologies, Carlsbad, CA, United States) using the FACS Aria II. RNA extraction was done using the Qiagen miRneasy Kit (Cat#217084, Qiagen, Hilden, Germany) and quality evaluated using an Agilent bio-analyzer instrument. cDNA was prepared using Clontech Ultra low input RNA kit v4 (Cat # 634888, Clontech, Mountain View, CA, United States), fragmented using Covaris and again evaluated for quality of using an Agilent bio-analyzer. Libraries were prepped using Clontech Low Input Library Rep Kit v2 (Cat # 634899, Clontech, Mountain View, CA, United States) and sequenced on an Illumina HiSeq 4000 (purchased from NIH funds under award number S10OD018220).

All data can be accessed from the Gene Expressions Omnibus^1^ using accession number GSE138882.

### Bulk RNA Sequencing Analysis

All analysis was performed using Galaxy^2^, an open source, web-based platform for data intensive biomedical research (Afgan et al., 2018)^3^. Analysis was performed on paired-ends fastqs using HISAT2 (Galaxy Version 2.2.1 + galaxy0) for alignment and read mapping to the mouse 10 mm reference genome yielding BAM files. StringTie (Galaxy Version 2.1.1) was used to assemble RNA-seq alignments into potential transcripts and DESeq2 (Galaxy Version 2.11.40.6 + galaxy2) to estimate differential expression of gene features from counts. Normalized count tables were used to visualize gene expression heatmaps for the top 100 genes and genes of interest.

### *In vitro* Tamoxifen Induction of *Actin^creERT2^;R26^Rainbow^* 2-D Suture Explants

Tamoxifen induction of 2-D suture explants was performed on calvarial sutures derived from postnatal day 3 *Actin^creERT2^;R26^Rainbow^* mice. 2-D suture explants were cultured under standard conditions as described above and in presence of 2.5 μM (Z)-4-hydroxytamoxifen (TAM) for 3 days, at day four the medium was replaced with fresh medium without TAM until explants were collected at day 10.

### Confocal Imaging

*Actin^creERT2^;R26^Rainbow^* 2-D suture explants images were captured using a LEICA TCS SP8 × laser scanning confocal microscope (LEICA Microsystems, Buffalo Grove, IL, United States) with an objective lens (×20 HC PL APO IMM CORR CS2, H2O/Glycerol/oil, N.A. 0.75). Raw image stacks were imported into ImageJ (NIH) for further analysis.

### Statistical Analysis

Data values are presented as mean ± SMD of independent experiments. Two-tailed student *t*-test were performed. A *p-value < 0.05 was considered statistically significant.

## Data Availability Statement

All data can be accessed from the Gene Expressions Omnibus (http://www.ncbi.nlm.nih.gov/geo/) using accession number GSE138882.

## Ethics Statement

The animal study was reviewed and approved by the Stanford University Animal Care and Use Committee guidelines.

## Author Contributions

NQ designed the experiments, performed the experiments, analyzed the data, and wrote the manuscript. SM designed the experiments, performed the experiments, analyzed the data, and wrote the manuscript. MG imaged the data. JH performed the experiments. ML edited the manuscript. All authors contributed to the article and approved the submitted version.

## Conflict of Interest

The authors declare that the research was conducted in the absence of any commercial or financial relationships that could be construed as a potential conflict of interest.

## Publisher’s Note

All claims expressed in this article are solely those of the authors and do not necessarily represent those of their affiliated organizations, or those of the publisher, the editors and the reviewers. Any product that may be evaluated in this article, or claim that may be made by its manufacturer, is not guaranteed or endorsed by the publisher.

## Footnotes

1
http://www.ncbi.nlm.nih.gov/geo/

2
usegalaxy.org

3
https://doi.org/10.1093/nar/gky379
