# Supplementary material for: Harnessing a Feasible and Versatile ex vivo Calvarial Suture 2-D Culture System to Study Suture Biology
Source: Front Physiol. 2022 Feb 10;13:823661. doi: 10.3389/fphys.2022.823661 (PMC8871685; doi:10.3389/fphys.2022.823661)
Supplement: Supplementary file 1 [file Table_1.pdf]

## Supplementary Table 1

| Gene | Accession Number | Species | Primer Sequence | Annealing Temp | Amplicon Size |
|---|---|---|---|---|---|
| | | | **RT-PCR Primers** | | |
| *Smad6* | NM_008542.3 | Mouse | Fwd: TCTGCTTCGGTGGATTGCAT | 57ºC | 172bp |
| | | | Rev: CTTGGATTTTGCACGCACGA | | |
| *Gapdh* | BC083080 | Mouse | Fwd: CGGCAAATTCAACGGCACAGTCAA | 60.5ºC | 424bp |
| | | | Rev: CTTTCCAGAGGGGCCATCCACAG | | |
| | | | **Genotyping Primers** | | |
| General CRE | | Mouse | Fwd: GCGGTCTGGCAGTAAAAACTATC | | |
| | | | Re: GTGAAACAGCATTGCTGTCACTT | | ~100 bp |
| | | | Fwd: CTAGGCCACAGAATTGAAAGATCT | | |
| | | | Rev:GTAGGTGGAAATTCTAGCATCATCC | | ~324 bp |
| | | | | | |
| | | | | | |
| *Twist-1* | | | Fwd: GGTTTCCGACTAGAGGTTTCC | *TouchdownTwist (tm1Bhr) alternate 2 | Wild Type: ~221 bp, M |
| | | | WT Fwd: ACTGTCTGGGTCGCTGTTG | Mutant: 500bp | |
| | | | Mut Rev: CCTTCTATCGCCTTCTTGACG | | |

**Table 1.** List of primers used for gene expression analysis and genotyping. Gene names, gene-accession number, primer sequence, annealing temperature for PCR and size of amplified product are reported.

## Supplementary Table 2

**Flow Cytometry**

| Antigen | Clone | Supplier | Catalog Number | Conjugate | Concentration in 100 μL (3x10$^6$ cells) |
|---|---|---|---|---|---|
| **Primary Antibodies** | | | | | |
| Tie2 | TEK4 | eBioscience | 14-5987-85 | Purifed | 5μg |
| CD45 | 30-F11 | BioLegend | 103109 | PE-Cy5 | 0.1μg |
| Ter119 | Terr-119 | Invitrogen | 15-5921-81 | PE-Cy5 | 0.1μg |
| Thy1.1 | HIS51 | eBioscience | 47-0900-82 | APC-eFluor 780 | 0.2μg |
| Thy1.2 | 53-2.1 | eBioscience | 47-0902-82 | APC-eFluor 780 | 0.2μg |
| Ly-51 | 6C3 | BioLegend | 108311 | Alexa647 | 0.5μg |
| CD105 | MJ7/18 | eBioscience | 13-1051-82 | Biotin | 1 μg |
| CD200 | OX-90 | BioLegend | 123802 | Purifed | 1 μg |
| CD51 | RMV-7 | BD Bioscience | 551187 | PE | 1 μg |
| **Secondary Antibodies** | | | | | |
| Streptavidin | n/a | eBioscience | 25-4317-82 | PE-Cy7 | 0.2μg |

**Table 2.** List of antibodies employed for FACS analysis. Name of each antibody, clone-derivation, vendors, catalogue number and concentration are listed.

# Supplementary Table 3

| | | | |
|---|---|---|---|
| Alpha-MEM GlutaMax | Gibco-Life Technologies | 32561102 | - |
| Performance FBSA18:D30 | Gibco-Life Technologies | A31605-01 | 10% |
| Pen Strep | Gibco-Life Technologies | 26140-079 | 1% |
| Ciprofloxacin HCL | bioWORLD | 40310031-3 | 0.10% |
| **Osteogenic Differentiation Media** | | | |
| Stem Pro Osteogenesis Differenti | Gibco - Thermo Fisher Scientific | A1007201 | - |
| Pen Strep | Gibco-Life Technologies | 26140-079 | 1% |
| Ciprofloxacin HCL | bioWORLD | 40310031-3 | 0.10% |
| **Chondrogenic Differentiation Media** | | | |
| Stem Pro Chondrogenesis Differ | Gibco - Thermo Fisher Scientific | A1007101 | - |
| Pen Strep | Gibco-Life Technologies | 26140-079 | 1% |
| Ciprofloxacin HCL | bioWORLD | 40310031-3 | 0.10% |
| **Cell Dissociation** | | | |
| Stem Pro Accutase | Gibco | A1110501 | - |
| TrypLE Express | Gibco | 12605-010 | 1:1 Dilution in PBS |

**Table 3.** Supplies for cell growth and differentiation. Name of cell-growth, osteogenic and chrondrogenic media, fetal bovine serum, antibiotics, cell-dissociation enzymes, their catalogue number and vendors are described.

## Supplementary Table 4

| Reagent | Supplier | Catalog Number | Working Concentration |
|---|---|---|---|
| **Small Molecules** | | | |
| SB431542 | Selleckchem.com | S1067 | 10µM |
| **Cre Induction** | | | |
| Tamoxifen | Sigma-Aldrich | T5648-1G | 2.5µM |
| DMSO | ATCC | 4-X | - |
| **Histology** | | | |
| Glutaraldehyde Solution (Grade II, 25% in H2O) | Sigma-Aldrich | G6257 | 0.20% |
| Formaldehyde Aqueous Solution (Paraformaldehyde Aqueo | ELECTRON MICROSCOPY SCIEN | 15710 | 0.4-4% |
| Tissue-Tek O.C.T Compound | VWR | 25608-930 | - |
| **Molecular Biology Reagents** | | | |
| TRIzol Reagent | ThermoFisher/Invitorgen | 15596026 | - |
| SuperScript III First-Strand Synthesis System | ThermoFisher/Invitorgen | 18080051 | - |
| KAPA2G Fast HotStart ReadyMix | KAPA BIOSYSTEMS | KK5609 | - |
| HotStarTaq Plus Master Mix Kit | QIAGEN | 203645 | - |
| **RNA-Sequencing Reagents** | | | |
| miRneasy Micro Kit | Qiagen | 217084 | - |
| Ultra low input RNA kit v4 | Clontech | 634888 | |
| Low Input Library Rep Kit v2 | Clontech | 634899 | |
| Recombinant Rnase inhibitor (RRI) | Clontech | 2313B | 4 units |
| Triton X-100 | ThermoFisher | 85111 | 0.10% |
| dNTP | ThermoFisher | 10297018 | 2.4mM |
| OligodT30VN (5′AAGCAGTGGTATCAACGCAGAGTAC | Integrated DNA Technology | - | 2.5µM |
| Smartscribe Reverse Transcriptase | Clontech | 639538 | 100 units |
| Betaine | Sigma-Aldrich | B0300-5VL | 1M |
| Template Switch Oligo (5′-AAGCAGTGGTATCAACGCA( | Qiagen | - | 1µM |
| 1X Kapa HiFi HotStart | KAPA BIOSYSTEMS | KK2602 | - |
| ISPCR primer (5′-AAGCAGTGGTATCAACGCAGAGT-3′ | Integrated DNA Technology | - | 0.1µM |
| Fragment Anakyzer High Sensitivity NGS 1-6000 Kit | Agilent | 474-0500 | - |

**Table 4.** Reagents Miscellaneous**.** List of reagents used for cell and molecular biology experiments, their working concentration, catalogue number and vendors are listed.
