# Supplementary figures and images for: Harnessing a Feasible and Versatile ex vivo Calvarial Suture 2-D Culture System to Study Suture Biology
Source: Front Physiol. 2022 Feb 10;13:823661. doi: 10.3389/fphys.2022.823661 (PMC8871685; doi:10.3389/fphys.2022.823661)

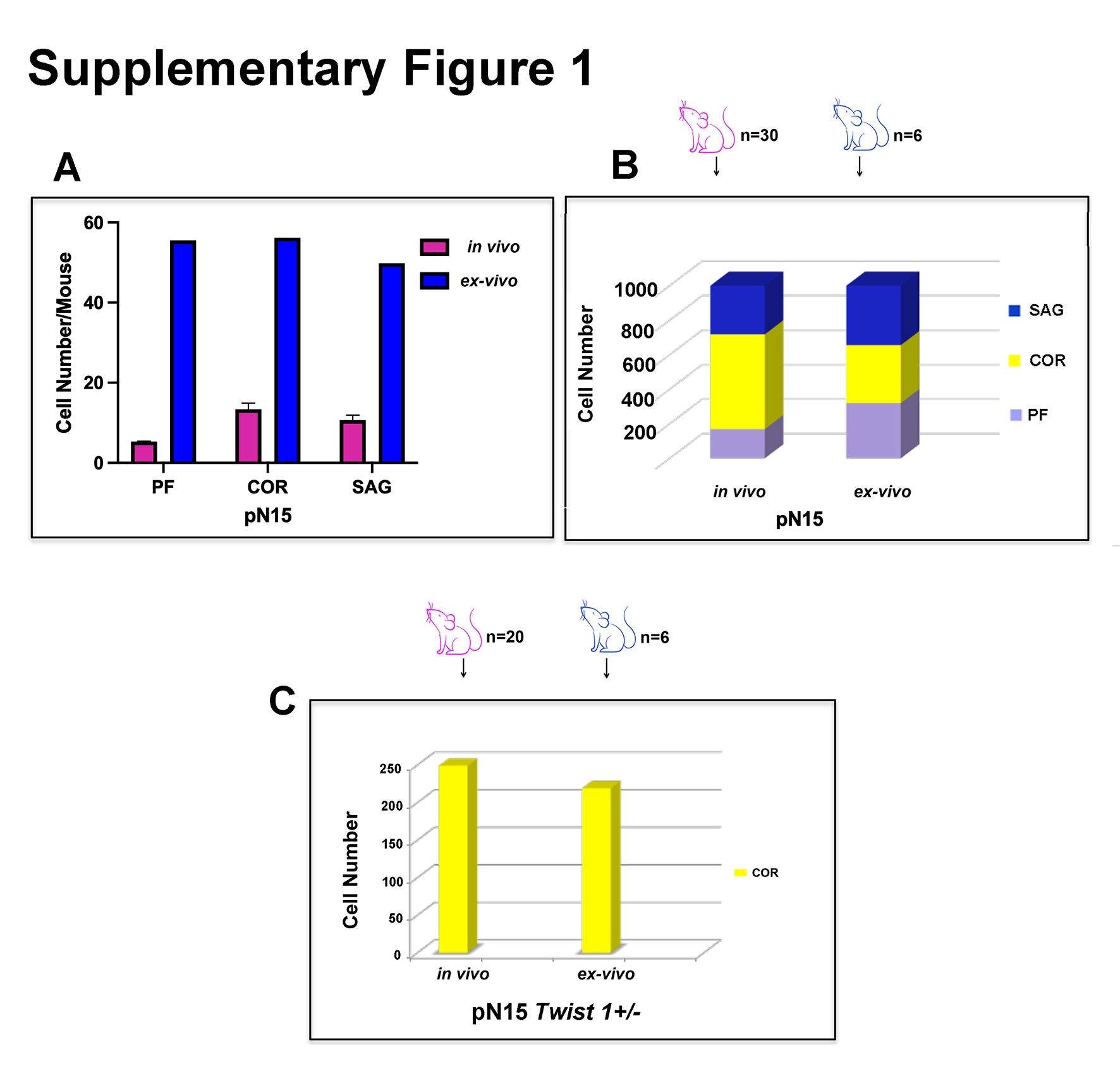

Supplement: Supplementary file 2 [file Image_1.TIF]
