# Supplementary material for: Harnessing a Feasible and Versatile ex vivo Calvarial Suture 2-D Culture System to Study Suture Biology
Source: Front Physiol. 2022 Feb 10;13:823661. doi: 10.3389/fphys.2022.823661 (PMC8871685; doi:10.3389/fphys.2022.823661)
Supplement: Supplementary file 3 [file Data_Sheet_1.docx]

**Supplementary Figure Legends**

**Supplementary Figure 1. Comparative analysis of skeletal stem/progenitor cells isolated from *in vivo* calvarial sutures and *ex-vivo* 2-D suture explants derived from pN15 CD-1 and *Twist 1* +/- mice. (A)** Histogram highlighting the remarkable difference in skeletal stem/progenitor cells representation between *in vivo* and *ex-vivo* sutures of pN15 mice. A dramatic reduction in *in vivo* representation of skeletal stem/progenitor cells as compared to *ex-vivo* suture explants is observed. Values are representative for mouse. **(B)** Histogram showing the higher yield of skeletal stem/progenitor cells isolated from *ex-vivo* 2-D suture explants using only six pN15 CD-1 mice in comparison to the *in vivo* procedure employing thirty mice.

**(C)** Histogram illustrating yields of skeletal stem/progenitor cells isolated from *Twist 1*^+/-^ mice employing *in vivo* and *ex-vivo* suture procedures. By using *ex-vivo* 2-D suture explants a number of only six mice was sufficient to provide a cell-yield close to that obtained by *in vivo* isolation from 20 mice.
